# Prenatal Exposure to Lead, δ-Aminolevulinic Acid, and Schizophrenia: Further Evidence

**DOI:** 10.1289/ehp.10464

**Published:** 2008-07-30

**Authors:** Mark G.A. Opler, Stephen L. Buka, Justina Groeger, Ian McKeague, Catherine Wei, Pam Factor-Litvak, Michaeline Bresnahan, Joseph Graziano, Jill M. Goldstein, Larry J. Seidman, Alan S. Brown, Ezra S. Susser

**Affiliations:** 1 Department of Psychiatry, College of Physicians and Surgeons, Columbia University, New York, New York, USA; 2 Department of Epidemiology, Brown University, Providence, Rhode Island, USA; 3 Department of Epidemiology, Columbia University, New York, New York, USA; 4 Department of Biostatistics, Mailman School of Public Health, Columbia University, New York, New York, USA; 5 New York State Psychiatric Institute, New York, New York, USA; 6 Department of Environmental Health Sciences, Mailman School of Public Health, Columbia University, New York, New York, USA; 7 Department of Psychiatry at Massachusetts Mental Health Center, Harvard Medical School, and Harvard Institute of Psychiatric Epidemiology and Genetics, Department of Medicine, Harvard Medical School, Boston, Massachusetts, and the Department of Women’s Health, Brigham and Women’s Hospital, Boston, Massachusetts, USA; 8 Public Psychiatry Division at Massachusetts Mental Health Center, and Department of Psychiatry, Massachusetts General Hospital, Harvard Medical School, Boston, Massachusetts, USA

**Keywords:** δ-aminolevulinic acid, developmental, lead, Pb, prenatal, prospective, psychosis, schizophrenia

## Abstract

**Background:**

A previously conducted study of prenatal lead exposure and schizophrenia using δ-aminolevulinic acid, a biologic marker of Pb exposure, in archived maternal serum samples collected from subjects enrolled in the Childhood Health and Development Study (1959–1966) based in Oakland, California, suggested a possible association between prenatal Pb exposure and the development of schizophrenia in later life.

**Objectives:**

In the present study we extend these findings using samples collected from the New England cohort of the National Collaborative Perinatal Project (1959–1966). Using similar methods, in this study we found results that suggest a comparable association in this cohort.

**Methods:**

We pooled matched sets of cases and controls from both the California and New England sites using a multilevel random-intercept logistic regression model, accounting for matching and site structure as well as adjusting for maternal age at delivery and maternal education.

**Results:**

The estimated odds ratio for schizophrenia associated with exposure corresponding to 15 μg/dL of blood Pb was 1.92 (95% confidence interval, 1.05–3.87; *p* = 0.03).

**Conclusion:**

Although several limitations constrain generalizability, these results are consistent with previous findings and provide further evidence for the role of early environmental exposures in the development of adult-onset psychiatric disorders.

A growing body of evidence supports the hypothesis that exposures that damage or disrupt the developing central nervous system are associated with schizophrenia and related disorders (McGrath and Murray 2003). Associations for some prenatal exposures have now been replicated—for example, nutritional deprivation (St. Clair et al. 2005; Susser et al. 1996). Exposure to potentially neurotoxic metals has not been carefully examined to date. In an earlier study, we measured a marker of second-trimester exposure to lead in prospectively collected serum samples from a birth cohort (Opler et al. 2004). The risk for schizophrenia spectrum disorders in adulthood was shown to be approximately doubled in subjects with maternal blood Pb levels (BPb) > 15 μg/dL [indicated by elevated levels of δ-aminolevulinic acid (δ-ALA)] during the second trimester, although the sample was too small to draw definitive conclusions and the results failed to reach statistical significance.

There are reasons to suspect that chemical agents in general, and particularly those associated with industrialization, may increase the risk of schizophrenia. Pb is a widely recognized developmental toxicant, leading to cognitive, behavioral, and physical impairments. Prospective birth cohorts in various parts of the world are consistent in finding that increased Pb exposure during development is associated with decrements in intellectual function up to 10 years of age [for a review, see Lanphear et al. (2005)]. There is also some suggestion that effects may persist into early adulthood; for example, Dietrich et al. (2001) found associations between elevated prenatal and childhood BPb concentrations and increased rates of delinquency during adolescence.

We designed the present study to expand our previous work with the Prenatal Determinants of Schizophrenia (PDS) study. As in our previous work, we capitalized on a large birth cohort in which pregnant women were recruited between 1959 and 1966. In this report, we used the New England sites of the National Collaborative Perinatal Project (NE-NCPP) (McGrath et al. 2003), whose data and design are similar to those of the PDS study. Many of the now adult offspring have been followed for neuropsychiatric outcomes. In this study, we independently analyzed these data, which we then pooled with data from our original work to test the hypothesis that prenatal Pb exposure is associated with risk of schizophrenia.

## Materials and Methods

### Description of cohorts

We obtained data and serum from two cohorts based in the United States. *a*) We included 81 subjects from the NE-NCPP. The NE-NCPP was part of a larger, multicenter cohort study initiated by the National Institute of Neurological and Communicative Disorders and Stroke, originally designed to identify associations between prenatal factors and adverse infant and child development. The New England cohorts comprise 17,741 pregnancies collected prospectively from 1959 to 1966 at the Child Study Center, Brown University, Providence, Rhode Island, and the Boston Hospital for Women and Children, Hospital Medical Center, Boston (McGrath et al. 2003). *b*) The PDS study is based on a cohort of live births collected prospectively from 1959 through 1967 at the Kaiser Foundation Health Plan clinics in Alameda County, California, as part of the Child Health and Development Study. The PDS study includes the 12,094 live-born individuals remaining in the health plan until 1981, when it became possible to use computerized records to identify potential cases of schizophrenia (Susser et al. 2000).

### Case ascertainment

**Figure 1:**
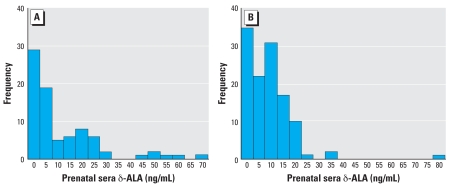
Serum δ-ALA distributions for NE-NCPP (mean ± SD = 11.6 ± 15.21; *n* = 81) (*A*) and PDS (mean ± SD = 9.0 ± 9.80; *n* = 119) (*B*) samples.

We identified individuals with major psychosis within the NE-NCPP cohorts through a two-stage diagnostic assessment procedure (Figure 1). In the first stage, we identified cohort members with possible psychotic illness through record linkages with public hospitals, mental health clinics, and the Massachusetts and Rhode Island Departments of Mental Health, and from personal interviews.

Using record linkage and personal interviews, we identified 241 potential cases. In the second stage, we located these 241 subjects through a variety of methods, including searches of credit bureaus, address directories, death certificates, motor vehicle reports, and home visits. Those who consented to participate in follow-up efforts were interviewed by a trained interviewer using the Structured Clinical Interview for the *Diagnostic and Statistical Manual for Mental Disorders*, 4th edition (*DSM-IV*) (American Psychiatric Association 2000), to determine lifetime prevalence of psychotic disorders as well as other mental disorders. Based on interview data and medical record review, trained PhD-and MD-level diagnosticians then completed best-estimate consensus diagnoses according to *DSM-IV* criteria. In the NE-NCPP, diagnostic interviews were completed for 167 subjects; medical charts alone were available for the remaining 74 subjects.

We drew the samples used in this study from subjects with schizophrenic psychoses and matched controls. Of the 241 potential subjects with psychosis, 111 subjects were determined to have a major psychotic disorder. Of these subjects, 51 were diagnosed with schizophrenic psychoses [schizophrenia (*n* = 47) or schizoaffective disorder (*n* = 4)]. A total of 27 cases (25 diagnosed with schizophrenia and 2 diagnosed with schizoaffective disorder) had serum samples available for this analysis. For each case subject, we selected two healthy controls matched for sex, race/ethnicity, and date of birth. We selected controls from an unaffected subset of subjects with no Axis I psychiatric diagnoses.

Overall, procedures for case ascertainment were similar to those used in the PDS study, including the use of consensus diagnostic procedures based on *DSM-IV* criteria. In the PDS study, a broader case definition was used for schizophrenia spectrum disorder, whereas the NE-NCPP included only schizophrenia and schizoaffective disorder. However, matching criteria for selecting controls in the NE-NCPP differed from the PDS study. Specifically, although both the NE-NCPP and PDS study matched on sex and date of birth, only the NE-NCPP matched on maternal ethnicity, and only the PDS study included the length of time subjects were in the cohort and the availability of maternal serum.

### Serum samples in the NE-NCPP

Women were registered in their first clinic or hospital visit, and in addition to detailed records on a variety of demographic characteristics of the parents and obstetric health, maternal blood was drawn every trimester and at birth (Buka et al. 2001). We obtained aliquots of 1 mL of maternal serum from both cases and controls, diluted 1:5 in phosphate-buffered saline and frozen at –20°C. We used third-trimester samples for 27 cases of schizophrenia spectrum disorder and 54 matched controls. Although seasonal variation has been noted to cause interindividual variation in third-trimester BPb levels, published data indicate that variability between the second- and third-trimester BPb levels is generally limited. However, there is some indication that Pb levels may increase toward the end of the third trimester. These fluctuations, when corrected using hematocrit levels to account for the increases in fluid volume, range from 0 to 1 μg/dL (Gulson et al. 2004).

### Laboratory protocol for NE-NCPP

Procedures for analysis of δ-ALA were similar to those used previously in analysis of samples from the PDS study. In brief, we thawed frozen serum samples and centrifuged them for 30 min at 14,000 rpm at 4°C. We removed an aliquot of supernatant and added it to 3.0 mL of acetylacetone reagent and 0.45 mL of 37% formaldehyde. Aliquot volumes ranged from 50 to 500 μL, based on dilution and starting volume. We loosely capped tubes and heated them on an aluminum alloy block heater in a darkroom for 40 min and then removed them from the heat to a test tube rack to cool in the darkroom at room temperature for 4 hr. We removed supernatant from the test tubes to Eppendorf tubes and centrifuged the tubes for 30 min in a 4°C cool room. We then filtered the supernatant and analyzed 750 μL using a PerkinElmer model LC-250 equipped with an LC-600 autosam-pler and an LC-40 fluorescence detector (PerkinElmer, Norwalk, CT) with separation conditions as previously reported.

### Exposure definition and statistical methods

We classified subjects according to previously validated methods, that is, having a BPb of ≥ 15 μg/dL or < 15 μg/dL, based on a cutoff of 9.05 ng/mL δ-ALA (Opler et al. 2004). As discussed in our prior publication (Opler et al. 2004), we found this method to have a moderate correlation (0.64) when used as a continuous measure, but when used categorically to predict BPb levels above or below 15 μg/dL, it was highly sensitive (90–91%) with positive predictive values of 89–91%. A calculated kappa statistic for duplicate samples was 0.89 with an SE of 0.23, indicating excellent agreement.

Using this method and exposure definition, we fitted conditional logistic regression models and estimated odds ratios (ORs) relating exposure status to a diagnosis of schizophrenia spectrum disorder. We combined results from the NE-NCPP with previously collected data from a nested sample of 119 subjects (44 cases and 75 controls) from the PDS study, described in detail elsewhere (Opler et al. 2004). The combined samples from the PDS study and NE-NCPP totaled 200 subjects (71 cases and 129 controls). To examine the relationship between δ-ALA and schizophrenia spectrum disorders in the combined samples, we used two approaches. First, we fitted conditional logistic regression models, including δ-ALA exposure as a predictor of schizophrenia spectrum disorder, while adjusting for covariates. Next, we fitted a multilevel random intercept logistic regression model using generalized linear latent and mixed models (GLLAMM) software within the STATA statistical package (StataCorp, College Station, TX) to account for the multilevel structure of the data (Rabe-Hesketh et al. 2004). Thus, we took into account the potential correlations within each “site” (i.e., PDS study vs. NE-NCPP).

We assessed potential confounders based on their known association with Pb exposure and/or schizophrenia, including maternal age at delivery, paternal age at delivery, parental employment status, maternal education, maternal race/ethnicity, and paternal education. A change of ±10% in the point estimate corresponding to the Pb exposure variable provided justification for including a variable in the model. Because we included maternal race/ethnicity in the matching criteria for the NE-NCPP sample, we used an indicator variable that coded for this covariate only in the PDS study.

## Results

These cohorts have previously been reported to differ in terms of several variables, including ethnicity, maternal age, and education (Strauss and Dietz 1999). The samples that were obtained for this study reflect these differences (Table 1). The NE-NCPP cohort was younger, was less ethnically diverse, and had a higher proportion of mothers who did not complete high school. By contrast, the PDS sample was more ethnically diverse, and most of the mothers in the sample had graduated high school and/or received further education.

### δ-ALA concentrations

As previously reported, retention times for δ-ALA occurred within a limited range, consistently near the mean (± SD) of 9.2 ± 0.36 min. Similar to previous findings, detectable δ-ALA peaks were very clear, and chromatograms were consistently characterized by low baselines and high signal-to-noise ratios. No other detectable compounds had retention times or signals that caused interference with the δ-ALA peak.

**Figure 2:**
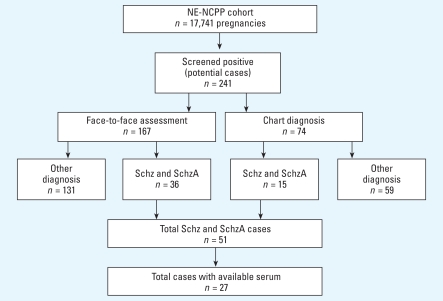
Case ascertainment in the NE-NCPP. Abbreviations: Schz, schizophrenia; SchzA, schizoaffective disorder.

In subjects from the NE-NCPP sample, δ-ALA levels ranged up to 70.5 ng/mL (Figure 2A), with a mean (± SD) of 11.6 ± 15.2 ng/mL; the mean concentration in the PDS study was 9.0 ± 9.8 ng/mL. Despite some differences in the distribution of δ-ALA concentrations, a similar proportion of subjects within each sample met the study defini-tion for Pb exposure (Figure 2B). We classified 42% of subjects (*n* = 34) as exposed in the NE-NCPP sample and 45% of subjects as exposed (*n* = 53) in the PDS sample.

### Distribution of covariates by exposure and case status

We noted differences in distributions of covariates between sites (Table 1). Although the NE-NCPP sample demonstrated no difference in maternal age in exposed versus unexposed subjects, in the PDS sample mothers of unexposed subjects were on average 1 year older than those classified as exposed. Mean maternal age was higher in cases than in controls at both sites, but this was not associated with risk of disease in either the NE-NCPP sample [OR = 1.01; 95% con-fidence interval (CI), 0.62–1.64; *p* = 0.97] or the PDS sample (OR = 1.1; 95% CI, 0.54–2.4; *p* = 0.8). Higher maternal age appears to be associated with lower exposure levels, although this association is not statistically significant for either the NE-NCPP sample (OR = 0.744; 95% CI, 0.25–2.23; *p* = 0.6) or the PDS sample (OR = 0.78; 95% CI, 0.43–1.41; *p* = 0.4). Higher maternal education (i.e., graduation from high school or equivalent) had a nonsignificant protective association with case status in the NE-NCPP sample (OR = 0.49; 95% CI, 0.17–1.38; *p* = 0.17) and PDS sample (OR = 0.49; 95% CI, 0.19–1.22; *p* = 0.12). Conversely, the risk of exposure appears to be higher among subjects whose mothers completed high school for both the NE-NCPP sample (OR = 2.08; 95% CI, 0.84–5.16; *p* = 0.11) and the PDS sample (OR = 1.43; 95% CI, 0.57–3.6; *p* = 0.43).

### Estimates of effect size

Table 2 presents the conditional results from independent and combined analyses, as both unadjusted and adjusted effect estimates. With conditional logistic regression, the unadjusted estimated OR for schizophrenia in subjects classified as exposed in the NE-NCPP cohort was 1.58 (95% CI, 0.55–4.51). As previously reported, a similar unadjusted measure in the PDS study yields an OR = 1.89 (95% CI, 0.86–4.11). After we combined both samples into a multilevel, random-intercept logistic regression model, the estimate of the unadjusted OR was 1.72 (95% CI, 0.96–3.09). When adjusted for maternal age and education, the estimated OR was 1.92 (95% CI, 1.05–3.52).

We tested several other covariates in the model including paternal age, paternal employment status, and maternal race/ethnicity. None of the additional variables tested had any appreciable impact, defined as a change of ±10% or more on the coefficient of the exposure variable when added to or removed from the model. In the saturated model, including all available covariates, the estimated OR for exposure was 1.96 (95% CI, 1.04–3.69).

## Discussion

We have attempted to build on our earlier study by first replicating our previous results in the NE-NCPP sample and then pooling subjects from two separate cohorts to increase our sample size. This new study expands on our preliminary work, further demonstrating a possible association between Pb exposure during development and schizophrenia. When we adjusted our findings for covariates, they provide support for previously reported results, with consistent effects across individual sites and in the pooled analysis. Despite some differences in sample and site characteristics, the effect sizes ranges from 1.6 to 1.9 (Table 2). In our samples, elevated prenatal levels of δ-ALA, the proxy for Pb exposure, is associated with almost a 2-fold increase in risk for schizophrenia spectrum disorder in adulthood.

Although these cohorts differed in some respects, there were remarkable similarities, including the birth years, comparability of serum storage procedures, and methods of case ascertainment. Nonetheless, our analysis faced challenges similar to those encountered in meta-analyses—for example, the need to pool results from different studies while taking into account the differences in source populations and methodologies. Although factoring in these differences, our analysis also had to preserve the matching structure of each study sample. The matching criteria for both samples were similar in some respects, but not identical: Most notably, the NE-NCPP was matched on race, whereas the PDS study was not. It was also extremely important for us not to use any method that would result in the loss of any subjects or matched sets, thus negating the use of extremely limited archived serum samples. The multilevel, random intercept logistic regression model that we finally employed met these requirements accounting for both site- and matched-set correlations.

A limitation of the previous study, which also applies here, is the use of δ-ALA to approximate Pb exposure. Because most of the Pb in blood is contained in erythrocytes, which are absent in sera, we have measured Pb exposure indirectly by quantifying the amount of δ-ALA in each serum sample. δ-ALA, a metabolite in the heme pathway, normally dimerizes rapidly in the presence of δ-ALA dehydrase to form porphobilinogen. However, in Pb-exposed individuals, δ-ALA levels in serum will be abnormally high because Pb, a potent inhibitor of erythrocyte δ-ALA dehydrase activity, prevents dimerization (Secchi et al. 1974). The laboratory methods we have used are adapted from standard techniques described by several researchers (Endo et al. 1993; Oishi et al. 1996) to determine levels of δ-ALA in plasma and further adapted by Tomokuni et al. (1993) for use on archived serum samples.

We found approximately the same percentage of exposed subjects in both samples, indicating that between 42% and 45% have estimated BPb levels of ≥ 15 μg/dL. This is consistent with the somewhat limited literature on BPb concentrations in women from samples that are contemporary with the NE-NCPP and PDS study. A study of proximity to freeways in Los Angeles County, California, found mean BPb levels of 16.7 ± 7.0 μg/dL for exposed women and 9.9 ± 4.9 μg/dL for unexposed women (Thomas et al. 1967). The NE-NCPP sample had slightly higher exposure levels than the PDS sample, as reflected in the slightly higher mean δ-ALA level. Although it might be suspected that this is attributable to older housing stock built before the 1950s, thus predating the removal of Pb-based paint, housing that is likely to contain Pb-based paint was prevalent (and continues to be so) both in New England and in Oakland, California. In particular, the city of Oakland and Alameda County are known to contain a very high percentage of houses with either exterior or interior Pb-based paint (Centers for Disease Control and Prevention 1992). The work of Meyer et al. (2003) shows that 50% of existing housing containing Pb-based paint within the United States was located in seven states—including California and Massachusetts. Other unmeasured variables could contribute to differences in exposure levels between the two sites, including the state of the home environment maintenance conditions (Sargent et al. 1997) and levels of automobile traffic (Rabinowitz et al. 1984), because these subjects were recruited before the ban on leaded gasoline.

More detailed information regarding certain covariates might have been beneficial, but it is doubtful whether their inclusion would have made a significant difference in the results. For example, more specific information on the level of exposure to urban environments (sometimes referred to as “urbanicity”) would have been desirable. Urbanicity is a suspected risk factor for schizophrenia as well as a determinant of Pb exposure (Pedersen et al. 2004). Although chemical exposures and ambient pollutants are more prevalent in urban settings (Marcelis et al. 1998), work by Pedersen and Mortensen (2006) suggests that the association between urbanicity and schizophrenia may not be mediated by exposure to toxins. A review by Krabbendam and van Os (2005) argues that because urban-environment–related cases of schizophrenia are not associated with neuropsychological impairments or obstetric complications, neurodevel-opmental mechanisms are less likely to be involved in the relationship.

Although we cannot entirely rule out the possibility that our estimates of prenatal Pb exposure are acting as a marker of the number of years spent in urban environments before pregnancy, there are several reasons that maternal urbanicity is not a likely factor in our results. Both the NE-NCPP and PDS samples are derived from cohorts based in urban centers. Consequently, the level of variability is likely to be limited, and very few subjects or parents within either sample are likely to be classified as anything but urban residents.

Parental socioeconomic status is another variable we would have liked to analyze in more detail. Like urban residence, it is also associated with elevated prenatal and postna-tal Pb exposure and has been associated with schizophrenia in some settings. However, when analyzed in our previous study, it proved to have no impact on the results (Opler et al. 2004). Other potential covariates that were unavailable for this analysis included family history of schizophrenia and obstetric complications.

Thus far, we have hypothesized that *in utero* exposure and subsequent changes in neurodevelopment were the most likely causal pathway to explain our results. Guilarte (2004) suggested that Pb exposure is a biologically plausible risk factor for schizophrenia that may act via the N-methyl D-aspartate (NMDA) receptor; indeed, Nihei and Guilarte (2001) demonstrated experimentally that Pb exposure may cause changes in the expression and function of the NMDA receptor. This receptor is particularly critical for learning and memory and synaptogenesis during development, and evidence implicating the role of NMDA and glutamate in schizophrenia has been mounting and has now led to the development of new psychopharmacologic agents (Imre 2007). Although our results provide additional support for the theory that Pb may exert developmental effects via changes in expression and function of NMDA receptor, new studies will be required to confirm our findings. Because *in utero* exposures are often correlated with exposures during the life course, these future studies will need to

As multiple lines of evidence from epidemiology converge, they may offer new targets for translational research. In our studies, we used serologic evidence to test the hypothesis that early developmental exposure to a known toxin was associated with elevated risk of psychotic disorders in later life. Biologic mechanisms consistent with our findings and with available epidemiologic and clinical evidence might be further investigated using animal models of exposure and disease, designed specifically to test potential common pathways for schizophrenia.

## Figures and Tables

**Table 1:** Demographic variables in the PDS and NE-NCPP samples.

Measure	PDS (%)^a^	NE-NCPP (%)^a^
Case status (no.)		
SSD^b^ cases	44	27
Matched controls	75	54
Maternal age (years)		
0–19	11.4	16
20–29	47.1	67.9
30–39	37	14.8
≥40	4.2	1.2
Race		
White	47.9	74.1
Black	40.3	25.9
Other	10.1	0
Paternal age (years)		
20–29	33.6	51.9
30–39	37.8	27.2
≥40	16.8	7.4
Mother’s education		
< High school	11.8	43.2
≥High school	79	54.
Unknown	8.4	2.5

a All values are percentages except for case status.

b Schizophrenia spectrum disorder, defined in the PDS study as schizophrenia, schizoaffective disorder, delusional disorder, schizoid personality disorder, or psychosis not otherwise specified, and in NE-NCPP as schizophrenia and schizoaffective disorder.

**Table 2:** Estimated ORs relating δ-ALA (categorized as ≥9.05 and < 9.05 ng/mL) and schizophrenia spectrum disorder using four statistical models.

Cohort	Cases	Controls	Model	Estimated OR (95% CI)	*p*-Value
NE-NCPP (Boston/Providence)	27	54	Conditional logistic regression, unadjusted for covariates	1.58 (0.55–4.51)	0.394
PDS (California)	44	75	Conditional logistic regression, unadjusted for covariates	1.89 (0.86–4.11)	0.109
PDS and NE-NCPP (combined)	71	129	Conditional logistic regression, unadjusted for covariates	1.77 (0.94–3.32)	0.074
			Random intercept logistic regression, unadjusted for covariates	1.72 (0.96–3.09)	0.071
			Conditional logistic regression, adjusted for maternal age and maternal education	2.17 (1.12–4.17)	0.020
			Random intercept logistic regression, adjusted for maternal age and maternal education	1.92 (1.05–3.52)	0.035
